# Resurgent and Gating Pore Currents Induced by *De Novo SCN2A* Epilepsy Mutations

**DOI:** 10.1523/ENEURO.0141-19.2019

**Published:** 2019-10-15

**Authors:** Emily R. Mason, Fenfen Wu, Reesha R. Patel, Yucheng Xiao, Stephen C. Cannon, Theodore R. Cummins

**Affiliations:** 1 Department of Pharmacology and Toxicology, Indiana University School of Medicine, Indianapolis, IN 46202; 2 Department of Physiology, David Geffen School of Medicine at the University of California at Los Angeles, Los Angeles, CA 90095-1751; 3 Program in Medical Neuroscience, Indiana University School of Medicine, Indianapolis, IN 46202; 4 School of Science, Department of Biology, Indiana University-Purdue University Indianapolis, Indianapolis, IN 46202; 5 School of Science, Biology Department Chair, Indiana University-Purdue University of Indianapolis, Indianapolis, IN 46202

**Keywords:** channelopathies, epilepsy, gating pore current, Nav1.2, resurgent current, whole-cell electrophysiology

## Abstract

Over 150 mutations in the *SCN2A* gene, which encodes the neuronal Nav1.2 protein, have been implicated in human epilepsy cases. Of these, R1882Q and R853Q are two of the most commonly reported mutations. This study utilized voltage-clamp electrophysiology to characterize the biophysical effects of the R1882Q and R853Q mutations on the hNav1.2 channel, including their effects on resurgent current and gating pore current, which are not typically investigated in the study of Nav1.2 channel mutations. HEK cells transiently transfected with DNA encoding either wild-type (WT) or mutant hNav1.2 revealed that the R1882Q mutation induced a gain-of-function phenotype, including slowed fast inactivation, depolarization of the voltage dependence of inactivation, and increased persistent current. In this model system, the R853Q mutation primarily produced loss-of-function effects, including reduced transient current amplitude and density, hyperpolarization of the voltage dependence of inactivation, and decreased persistent current. The presence of a Navβ4 peptide (KKLITFILKKTREK-OH) in the pipette solution induced resurgent currents, which were increased by the R1882Q mutation and decreased by the R853Q mutation. Further study of the R853Q mutation in *Xenopus* oocytes indicated a reduced surface expression and revealed a robust gating pore current at negative membrane potentials, a function absent in the WT channel. This not only shows that different epileptogenic point mutations in hNav1.2 have distinct biophysical effects on the channel, but also illustrates that individual mutations can have complex consequences that are difficult to identify using conventional analyses. Distinct mutations may, therefore, require tailored pharmacotherapies in order to eliminate seizures.

## Significance Statement

This study expands our understanding of the pathogenic biophysical effects associated with two common epilepsy mutations in the human neuronal voltage-gated sodium channel, hNav1.2. We show that different point mutations in hNav1.2 that are implicated in epilepsy syndromes have distinct biophysical effects on the channel, some of which are not revealed by standard electrophysiological analyses. The data herein suggests that alterations in resurgent currents and/or the creation of a gating pore, which is distinct from the central pore, may be involved in the mechanisms by which hNav1.2 mutations contribute to epilepsy and other problems. Uncovering the distinct mechanisms by which different mutations contribute to aberrant electrical activity will help us to develop more effective and personalized therapies for epileptic patients.

## Introduction

Over 150 mutations in the *SCN2A* gene, which encodes the neuronal voltage-gated sodium channel Nav1.2, have been implicated in human epilepsy cases, but few of them have been studied *in vitro* to determine the molecular basis of their pathogenicity. Many of these epilepsy cases are refractory to current pharmacological interventions, and understanding the molecular mechanisms by which these mutations cause epileptiform activity in the brain can help us find more effective pharmacological treatments.

Mutations in *SCN2A* are thought to cause epileptic seizures by allowing an excess of sodium to enter affected neurons, causing the neurons to aberrantly fire action potentials. This can be caused by shifts in gating parameters (e.g., the voltage dependences of activation and inactivation), altered channel conductance, altered expression of the channels in the neuronal membrane, or increases in auxiliary currents such as persistent currents. Augmentation of persistent currents, which are small currents that persist after most of the sodium channels have inactivated, is often accompanied by augmentation of resurgent current, which occurs after the channels typically are inactivated (Theile et al., 2011; Barker et al., 2017). This concurrent augmentation has been seen in hippocampal neurons after induction of epilepsy in three rodent models (Hargus et al., 2013; Barker et al., 2017; Ottolini et al., 2017; Shao et al., 2017) and is associated with neuronal hyperexcitability and repetitive firing (Raman and Bean, 1997; Khaliq et al., 2003; Afshari et al., 2004; Yue et al., 2005; Van Drongelen et al., 2006; Kim et al., 2010; Hargus et al., 2013). Missense mutations at arginines of the S4 segments in the voltage sensor domains may produce an anomalous ion conduction pathway that supports a so-called gating pore current, also called omega current (Starace and Bezanilla, 2001, 2004; Tombola et al., 2005). These voltage-dependent sustained leak currents have been established as a cause of the skeletal muscle disorder hypokalemic periodic paralysis (Sokolov et al., 2007; Struyk and Cannon, 2007), where 19 of 20 reported mutations are R/X substitutions in S4 of the skeletal muscle sodium channel, Nav1.4, or of the muscle L-type Ca channel, Cav1.1 (Cannon, 2018). Subsequently, R/X mutations of S4 in the cardiac sodium channel, Nav1.5, associated with dilated cardiomyopathy and arrhythmias have been shown to support gating pore current (Gosselin-Badaroudine et al., 2012; Moreau et al., 2015). While site-directed mutagenesis has demonstrated that the Nav1.2 neuronal sodium channel is capable of conducting gating pore current (Sokolov et al., 2005), to date, there has not been a report of a disease-associated R/X mutation in Nav1.2 that supports a gating pore current.

**Figure 1.**
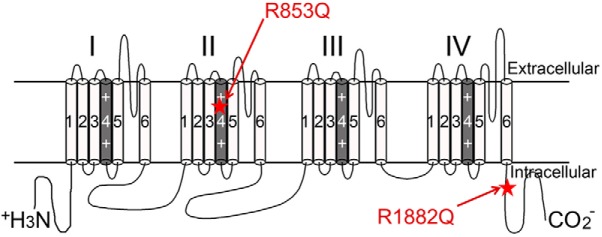
Mutation locations. Secondary structure of hNav1.2 is shown. Locations of the R853Q and R1882Q mutations are labeled with red stars and red arrows.

Among the Nav1.2 mutations suspected to cause epilepsy in human patients with otherwise idiopathic epileptic encephalopathies, R1882Q and R853Q are two of the most commonly reported mutations (Carvill et al., 2013; Epi4k Consortium, 2013; Nakamura et al., 2013; Howell et al., 2015; Samanta and Ramakrishnaiah, 2015; Kobayashi et al., 2016; Li et al., 2016; Trump et al., 2016; Wolff et al., 2017). The R1882 residue is located in the C-terminal tail of the channel, whereas R853 is a voltage-sensing residue in the second domain of the channel (Fig. 1). The seizures resulting from the R853Q mutation are typically refractory to the currently available antiepileptic drugs, including sodium channel blockers that are effective in suppressing seizures generated by other *SCN2A* mutations, including R1882Q, which suggests that the pathogenic biological effects of the R853Q mutation differ from those of the R1882Q mutation. In this study, we examined the effects of these two mutations on the function of human Nav1.2 proteins expressed in HEK cells, to identify mechanisms by which these mutations may be pathogenic. We validated some of the recent findings regarding these mutations using a different cell model (Berecki et al., 2018) and investigated the effects of these mutations on resurgent currents and gating pore currents, which the referenced study did not fully investigate. In transiently transfected HEK cells, the R1882Q mutation produced only gain-of-function effects, including robust resurgent currents, while the R853Q mutation produced mostly loss-of-function effects. However, we show that, when expressed in *Xenopus* oocytes, the R853Q mutation creates a gating pore, an anomalous gain, through which small amounts of current can flow when the cell membrane is hyperpolarized. The results of this study support the hypothesis that the R1882Q mutation increases neuronal excitability. It is not yet known how the loss-of-function effects of the R853Q mutation and the gating pore currents it causes might combine to lead to the initiation of seizures and other neurologic sequalae, but we discuss a few possibilities.

## Materials and Methods

### DNA constructs

Codon-optimized human Nav1.2 DNA constructs [wild-type (WT), R1882Q, and R853Q] were designed in-house and synthesized by GenScript. The amino acid sequence for the synthesized WT hNav1.2 cDNA construct corresponds with NG_008143.1 in the NCBI database. Synthesized mutant constructs are identical to WT aside from the single mutation (R1882Q or R853Q).

### HEK cell culture and transient transfections

The use of HEK293T cells (hereafter referred to as HEK cells; Dubridge et al., 1987) was approved by the Institutional Biosafety Committee and followed the ethical guidelines for the National Institutes of Health for the use of human-derived cell lines. HEK cells were grown under standard tissue culture conditions (5% CO_2_, 37°C) in maintenance media consisting of DMEM supplemented with 10% fetal bovine serum and 1% penicillin/streptomycin. HEK cells were transfected using the calcium phosphate precipitation method. Briefly, calcium phosphate-DNA mixture [2-μg channel construct and 1-μg enhanced green fluorescent protein (EGFP)] was added to cells in serum-free media for 8–17 h, after which the cells were washed with maintenance media and the serum-free media was replaced with maintenance media. Transfected cells were identified by presence of fine particulate coating the cells before washing and by expression of EGFP using a fluorescent microscope. Whole-cell patch clamp recordings were obtained 36–48 h after transfection.

### HEK cell electrophysiology

Currents were measured at room temperature (∼22°C) using a HEKA EPC 10 amplifier and the Pulse software (v8.80, HEKA Elektronik) for data acquisition. Electrodes were fabricated from 100-μl calibrated pipettes (Drummond Scientific Company; catalog #2-000-100; capillary glass) and fire-polished to a resistance of 1.0–2.0 MΩ using a P-1000 micropipette puller (Sutter Instrument Company). For each cell, a GΩ seal was obtained before breaking into the whole-cell configuration. Voltage protocols were initiated 5 min after entering the whole-cell configuration, allowing time for each cell’s cytoplasm to equilibrate with the pipette solution, while also controlling for time-dependent shifts in sodium channel properties. Voltage errors were minimized by using series resistance compensation (up to 90%), and passive leak currents were cancelled by P/-5 subtraction. The bath solution contained the following: 140 mM NaCl, 3 mM KCl, 1 mM MgCl_2_, 1 mM CaCl_2_, and 10 mM HEPES, adjusted to pH 7.30 with NaOH. The pipette solution contained the following: 130 mM CsF, 10 mM NaCl, 10 mM HEPES, and 1 mM CsEGTA, adjusted to pH 7.30 with CsOH. Navβ accessory subunits were not co-transfected with the Nav1.2α subunit, which functions independently as a channel, due to the variability that this would introduce into the experiments and the lack of information regarding which Navβ subunits are associated with the Nav1.2α subunits in the affected neurons in the brain. Instead, Navβ4 peptide (KKLITFILKKTREK-OH, used at 200 μM; Biopeptide Co., Inc.), which corresponds to part of the C-terminal tail of the full-length Nav β4 subunit, was included in the pipette solution to induce/amplify the resurgent currents. This peptide has been shown to induce resurgent currents in HEK cells expressing voltage-gated sodium channels, while, for unknown reasons, the full-length Navβ4 peptide is not sufficient to produce resurgent currents in HEK cells expressing voltage-gated sodium channels (Chen et al., 2008; Aman et al., 2009).

### HEK cell voltage protocols

All HEK cells were held at –100 mV.

**Figure 2.**
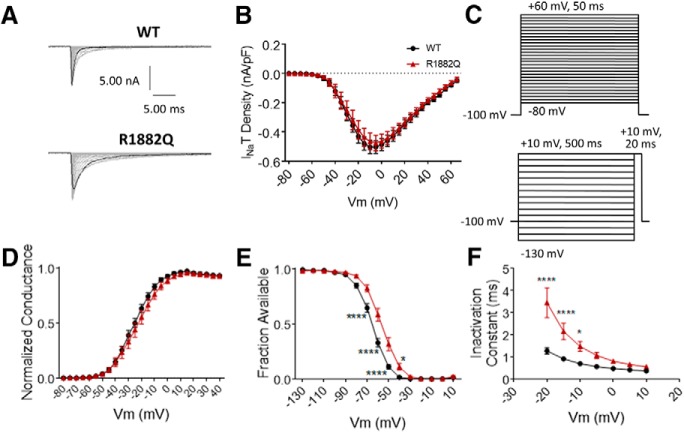
The R1882Q mutation has no effect on transient current size and impairs inactivation. ***A***, Representative raw current trace families elicited by voltage steps from –80 to +60 mV. ***B***, Average transient current densities elicited by voltage steps from –80 to +60 mV (*n* = 40 WT cells, *n* = 25 R1882Q cells). ***C***, Voltage stimulation protocols used for collection of activation (top) and inactivation (bottom) data from HEK cells. ***D***, Activation curves. Conductance was calculated as G/G_max_, where G = I/(V_m_-V_rev_), V_rev_ = reversal potential, and G_max_ = maximum inward conductance across all tested voltages (*n* = 41 WT cells, *n* = 27 R1882Q cells). ***E***, Inactivation curves. Fraction available was calculated as I/I_max_ for each cell at each voltage step (*n* = 40 WT cells, *n* = 23 R1882Q cells). ***F***, Time constants of fast inactivation were calculated by fitting the decay portion of each current trace to a single-exponential equation in PulseFit (HEKA; *n* = 41 WT cells, *n* = 27 R1882Q cells). WT data are represented by black circles, and R1882Q data by red triangles, in ***B***, ***D–F***. Error bars (SEM) are included for every data point in parts ***B***, ***D–F***; please note that many error bars are small and are thus obscured by the symbols. Asterisks indicate significance level (*p < 0.05, ****p < 0.0001). *Figure Contributions*: Emily Mason performed data collection and analysis.

Measures pertaining to transient current size, voltage-dependent activation, and persistent current were taken from a voltage protocol consisting of 50-ms test pulses to voltages from –80 to +60 mV, in 5-mV steps (Fig. 2*C*, top). Peak current amplitudes, for each voltage, were measured as the minimum value of the current over the entirety of the test pulse. Current densities were calculated by dividing raw current amplitudes by the membrane capacitance value of each cell. The reversal potential was estimated, for each cell, by extrapolation of the ascending limb of the current−voltage (IV) curve. The conductance values were then calculated and normalized within each cell. Persistent currents were measured, in Pulsefit (v8.80, HEKA Elektronik), as an average of the current amplitude over the last 10% of the test pulse, from 40 to 50 ms.

Inactivation was measured by a 500 ms prepulse step to voltages from –130 to +10 mV, in 10-mV steps, followed by a 20-ms test pulse to +10 mV (Fig. 2*C*, bottom). As with the activation protocol, peak transient current amplitude, for each voltage, was measured as the minimum value of the current over the entirety of the test pulse.

**Figure 3.**
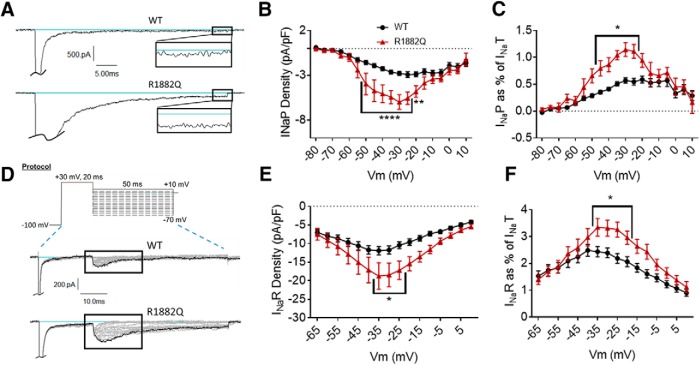
The R1882Q mutation enhances persistent and resurgent currents. ***A***, Representative raw current traces from a voltage step to –15 mV. Persistent current (box and inset) was measured as the average current over the last 10% (i.e., 5 ms) of the voltage step. ***B***, Average persistent current densities during the last 10% of voltage steps from –80 to +60 mV (*n* = 39 WT cells, *n* = 25 R1882Q cells). ***C***, Average persistent current amplitudes normalized to maximum peak transient current amplitudes (*n* = 40 WT cells, *n* = 27 R1882Q cells). ***D***, Resurgent current voltage protocol (top) and representative resulting raw current trace families (middle and bottom). ***E***, Average peak resurgent current densities over a range of voltages from –80 to +60 mV (*n* = 39 WT cells, *n* = 25 R1882Q cells). ***F***, Average peak resurgent current amplitudes normalized to maximum peak transient current amplitudes (*n* = 39 WT cells, *n* = 25 R1882Q cells). For ***B***, ***C***, ***E***, ***F***, values were calculated for each individual cell and averaged for each group. WT data are represented by black circles, and R1882Q data by red triangles, in ***B***, ***C***, ***E***, ***F***. Error bars (SEM) are included for every data point in parts ***B***, ***C***, ***E***, ***F***; please note that many error bars are small and are thus obscured by the symbols. Asterisks indicate significance level (*p < 0.05, **p < 0.01, ****p < 0.0001). *Figure Contributions*: Emily Mason performed data collection and analysis.

Resurgent currents were elicited by a 20-ms pulse to +30 mV, followed by a 50-ms repolarization step of voltages from +10 to –65 mV (Fig. 3*D*, top). The resurgent current peak amplitude was measured, in Pulsefit, as the minimum value of the current elicited during the repolarization step.

### Subcloning and expression in oocytes

For expression of hNav1.2 in oocytes, the *SCN2A* gene was cut from the pcDNA3.1 vector using the restriction enzymes NotI and NheI and subcloned into the pGEMHE-membrane-mEGFP plasmid (a gift from Melina Schuh, Addgene plasmid #105526; http://n2t.net/addgene:105526; RRID:Addgene_105526; Clift et al., 2017), replacing the EGFP gene with the *SCN2A* gene. The human β1 subunit, also in pGEMHE (Struyk and Cannon, 2007), was co-expressed with hNav1.2 because it normalizes the inactivation kinetics of voltage-gated sodium channels expressed in *Xenopus* oocytes (Patton et al., 1994). Complementary RNAs were synthesized *in vitro* with the mMessage mMachine transcription kit (Ambion). Oocytes were injected with ∼50 ng of the hNav1.2 WT or R853Q transcript plus 50 ng of the β1 subunit (four-fold molar excess).

### Oocyte maintenance

Oocytes were harvested from three female *Xenopus laevis* under MS222 anesthesia, in accordance with the guidelines established by the University of California, Los Angeles animal care committee’s regulations. After defolliculation in collagenase Type I at room temperature for ∼2 h, oocytes were injected with cRNA and stored at 18°C in 0.5 × Leibovitz’s L-15 medium (Gibco) supplemented with 1% horse serum, 100 U/ml penicillin, 100 μg/ml streptomycin, and 100 μg/ml amikacin.

### Oocyte electrophysiology

Currents were recorded 3–6 d after injection using a cut-open oocyte voltage-clamp with the CA-1B amplifier (Dagan Corp.) in headstage clamp mode, as previously described (Struyk and Cannon, 2007). The extracellular solution (upper and middle chambers) contained the following: 115 mM Na-methanesulfonate, 3 mM K-methanesulfonate, 4 mM Ca-acetate, 10 mM HEPES, 0.1 mM ouabain, and 0.005 mM tetrodotoxin (TTX), pH 7.4 with NaOH. The internal solution (lower chamber) contained the following: 120 mM K-methanesulfonate, 10 mM EGTA, and 10 mM HEPES, pH 7.4 with methanesulfonic acid. The lower surface of the oocyte was permeabilized by brief exposure to internal solution supplemented with 0.1% saponin.

### Oocyte voltage protocol(s)

To record gating pore current, an oocyte was held at –100 mV and a series of 200-ms voltage steps from –140 to +40 mV was applied. No on-line leak subtraction was used. Instead, a linear fit of the (total) steady-state IV for test potentials from –20 to +10 mV obtained to estimate the nonspecific linear current, which was subtracted to obtain the gating pore current. Charge displacement current was recorded by application of a series of 20-ms test depolarizations from –140 to +40 mV from a holding potential of –100 mV. The membrane capacitance was analog compensated with the amplifier circuitry, and the residual linear leakage current suppressed by P/N on-line leak subtraction of the current elicited by a depolarization from –120 to –100 mV. The charge displacement current was integrated digitally to obtain on-gating charge, Q_on_.

### Statistics and analysis

Electrophysiological data were analyzed using Pulsefit (v.8.80, HEKA Elektronik), Microsoft Excel, Origin (v9.1, OriginLab Corp.), and GraphPad Prism (v.7.03, GraphPad Software, Inc.). All data points are presented as mean ± SEM, and *n* is the number of cells from which contributing data were collected. Current density and conductance were calculated as previously described (Cummins et al., 1994; also, see above, HEK cell voltage protocols). Activation and inactivation midpoints were estimated by fitting the conductance and inactivation curves to a Boltzmann equation in Pulsefit. Inactivation time constants were estimated by fitting the decay portion of each sodium current trace to a single-exponential equation in Pulsefit.

The normality of data distribution was evaluated with the Shapiro–Wilk normality test. If the data were normally distributed, a parametric test was used; if the data were not normally distributed, a nonparametric test was used. For data comparisons spanning multiple voltages, a two-way ANOVA with the Sidak’s multiple comparisons test was used. When a single measure was compared between the WT and mutant groups, the data were compared with one-way ANOVAs. If the data were normally distributed, an ordinary one-way ANOVA with Dunnett’s multiple comparisons test was used; if the data were not normally distributed, the nonparametric Kruskal–Wallis with Dunn’s multiple comparisons test was used. Statistical significance was set at α = 0.05. Unless otherwise stated, *p* values reported for ANOVAs are the *p* values obtained from the *post hoc* test, and a significant *p* value (*p* < 0.05) was obtained in the ANOVA test. Specific statistical results are shown in Table 1. For all figures, *p < 0.05, **p < 0.01, ***p < 0.001, and ****p < 0.0001.

**Table 1.** Statistical table

Figure	Data type	*N* (group)	Statistical test(s)	Power/significance
2*B*	Each data point represents the group average value at that particular membrane potential	40 (WT cells), 25 (R1882Q cells)	Two-way ANOVA, Dunnett’s multiple comparisons test	Column factor *p* < 0.0001, WT vs R1882Q, *p* > 0.40 at all tested membrane potentials
2*D*	Each data point represents the group average value	41 (WT cells), 27 (R1882Q cells)	Two-way ANOVA, Dunnett’s multiple comparisons test	Column factor *p* = 0.0004, WT vs R1882Q, *p* > 0.40 at all tested membrane potentials
2*E*	Each data point represents the group average value	40 (WT cells), 23 (R1882Q cells)	Two-way ANOVA, Dunnett’s multiple comparisons test	Column factor *p* < 0.0001; WT vs R1882Q *p* = 0.0891 at –80 mV, *p* = 0.0001 from –70 to –50 mV, *p* = 0.0398 at –40 mV
2*F*	Each data point represents the group average value	41 (WT cells), 27 (R1882Q cells)	Two-way ANOVA, Dunnett’s multiple comparisons test	Column factor *p* < 0.0001, WT vs R1882Q *p* < 0.0001 at –20 and –15 mV, *p* = 0.0185 at –10 mV
3*B*	Each data point represents the group average value	39 (WT cells), 25 (R1882Q cells)	Two-way ANOVA, Dunnett’s multiple comparisons test	Column factor *p* < 0.0001, WT vs R1882Q *p* = 0.0001 from –50 to –25 mV, *p* = 0.0016 at –20 mV, *p* = 0.0526 at –15 mV
3*C*	Each data point represents the group average value	40 (WT cells), 27 (R1882Q cells)	Two-way ANOVA, Dunnett’s multiple comparisons test	Column factor *p* < 0.0001, WT vs R1882Q *p* = 0.0003–0.017 from –45 to –25 mV
3*E*	Each data point represents the group average value	39 (WT cells), 25 (R1882Q cells)	Two-way ANOVA, Dunnett’s multiple comparisons test	Column factor *p* < 0.0001, WT vs R1882Q, *p* = 0.0084 – 0.0466 from –35 to –20 mV
3*F*	Each data point represents the group average value	39 (WT cells), 25 (R1882Q cells)	Two-way ANOVA, Dunnett’s multiple comparisons test	Column factor *p* < 0.0001, WT vs R1882Q, *p* = 0.0053 – 0.0446 from –35 to –20 mV
4*B*	Each data point represents the group average value	40 (WT cells), 16 (R853Q cells)	Two-way ANOVA, Dunnett’s multiple comparisons test	Column factor *p* < 0.0001; WT vs R853Q *p* = 0.0250 (*) at –30 mV, *p* = 0.0037 (**) at –25 mV, *p* = 0.0002 (***) at –20 mV, *p* = 0.0001 (****) from –15 to +5 mV, *p* = 0.0005 (***) at +10 mV, *p* = 0.0006 (***) at +15 mV, *p* = 0.0056 (***) at +20 mV, *p* = 0.0160 (*) at +25 mV, *p* = 0.0293 (*) at +30 mV, *p* = 0.0733 (ns) at +35 mV
4*D*	Each data point represents the group average value	41 (WT cells), 16 (R853Q cells)	Two-way ANOVA, Dunnett’s multiple comparisons test	Column factor *p* = 0.0004, WT vs R853Q, *p* > 0.99 at all tested membrane potentials
4*E*	Each data point represents the group average value	40 (WT cells), 16 (R853Q cells)	Two-way ANOVA, Dunnett’s multiple comparisons test	Column factor *p* < 0.0001, WT vs R853Q, *p* = 0.0001 at –70 and –60 mV
4*F*	Each data point represents the group average value	41 (WT cells), 16 (R853Q cells)	Two-way ANOVA, Dunnett’s multiple comparisons test	Column factor *p* < 0.0001, WT vs R853Q *p* > 0.99 at all tested membrane potentials
5*B*	Each data point represents the group average value	39 (WT cells), 16 (R853Q cells)	Two-way ANOVA, Dunnett’s multiple comparisons test	Column factor *p* < 0.0001, WT vs R853Q *p* = 0.0028 – 0.0500 from –35 to +5 mV, *p* = 0.0670 (ns) at +10 mV
5*C*	Each data point represents the group average value	40 (WT cells), 16 (R853Q cells)	Two-way ANOVA, Dunnett’s multiple comparisons test	Column factor *p* < 0.0001, WT vs R853Q *p* > 0.10 at all tested membrane potentials
5*E*	Each data point represents the group average value	39 (WT cells), 16 (R853Q cells)	Two-way ANOVA, Dunnett’s multiple comparisons test	Column factor *p* < 0.0001, WT vs R853Q *p* = 0.0058 – 0.0137 from –40 to –30 mV
5*F*	Each data point represents the group average value	39 (WT cells), 16 (R853Q cells)	Two-way ANOVA, Dunnett’s multiple comparisons test	Column factor *p* < 0.0001, WT vs R853Q *p* = 0.0972 at –35 mV, *p* > 0.10 at all tested membrane potentials
6*C*	Each data point represents the group average value	5 (WT cells), 8 (R853Q cells)	Two-way ANOVA with Sidak’s multiple comparisons test	Column factor *p* < 0.0001; multiple comparisons *p* = 0.0745 at –20 mV, *p* = 0.0026 at –10 mV, *p* = 0.0001 at 0 mV, *p* < 0.0001 from 10 to 40 mV
6*D*	Each data point represents the group average value	5 (WT cells), 8 (R853Q cells)	Two-way ANOVA with Sidak’s multiple comparisons test	Column factor *p* = 0.1488
7*A–F*	Representative single-cell data	1 (WT cell), 1 (R853Q cell)	No statistical tests performed	
8	Representative single-cell data	1 (WT cell), 1 (R853Q cell)	No statistical tests performed	
9*A*	Each data point represents values from a single cell	6 (WT cells), 11 (R853Q cells)	Linear correlation analysis, constrained through origin	R853Q: slope = –118 nC/nA, Pearson’s *R* = –0.974, *R* ^2^ (adjusted) = 0.943
9*B*	Each data point represents the group average value	6 (WT cells), 11 (R853Q cells)	Two-way ANOVA with Sidak’s multiple comparisons test	Column factor *p* < 0.0001; multiple comparisons *p* < 0.0001 from –120 to –70 mV, *p* = 0.0002 at –60 mV, *p* = 0.0359 at –50 mV; *p* > 0.5000 for all other membrane potentials

When data for the R1882Q mutant, the R853Q mutant, and the WT channel were collected concurrently, ANOVA or nonparametric tests were used to analyze the HEK cell data. The values for each mutant were compared to the WT channel values, and thus Dunn’s or Dunnett’s *post hoc* tests were used to determine significant differences between mutant and WT channel values. Data from oocytes only included two groups, WT and R853Q, and therefore a nonparametric Mann–Whitney test was used to compare Q_max_ values obtained from oocytes.

## Results

### Effects of the R1882Q mutation on channel function

#### The R1882Q mutation impairs fast inactivation

To investigate the functional effects of the R1882Q mutation on hNav1.2 channel function, the biophysical properties of WT and R1882Q mutant voltage-gated sodium channels were characterized using whole-cell voltage clamp electrophysiology. The channel constructs were transiently expressed in HEK cells, which we have found to express little to no endogenous voltage-gated sodium current (–207.1 ± 33.64 pA, *n* = 10). Therefore, most of the voltage-gated inward current that we see, which, on average, is many times the amplitude of the average endogenous current, can be attributed to the WT or R1882Q channels for which the DNA was transfected.

The R1882Q mutation had no effect on the average peak transient current density elicited by voltage steps between –80 and +65 mV (Fig. 2*A*,*B*, *p* > 0.40 at all tested membrane potentials). It also had no effect on the average maximum peak transient current amplitude (an average of the maximum peak transient current amplitude values for individual cells, *n* = 41 WT, *n* = 27 R1882Q; *p* > 0.99), or the average maximum peak transient current density (an average of the maximum peak transient current density values for individual cells, *n* = 40 WT, *n* = 22 R1882Q; *p* = 0.80) when transiently expressed in HEK cells. Thus, the R1882Q mutation does not alter transient hNav1.2 current amplitudes or densities in HEK cells.

The voltage dependence of activation and inactivation were assessed using step depolarizations as seen in the voltage protocols (Fig. 2*C*). The R1882Q mutation produced no significant shift in the conductance curve of Nav1.2, compared to WT (Fig. 2*D*, *p* > 0.40 at all tested membrane potentials) and had no effect on the average midpoint of activation (WT –26.50 ± 1.48 mV, R1882Q –24.77 ± 2.76 mV, *n* = 41 WT, *n* = 24 R1882Q; one-way ANOVA *p* = 0.79). The R1882Q mutation produced a significant depolarizing shift in the fast inactivation curve (Fig. 2*E*, *p* = 0.0001 from –70 to –50 mV, *p* = 0.040 at –40 mV), which was reflected in the depolarization of the average inactivation midpoint (WT –65.01 ± 1.27 mV, R1882Q –57.34 ± 2.05 mV, *n* = 40 WT, *n* = 20 R1882Q; *p* = 0.0017). The R1882Q mutation also slowed inactivation, as evidenced by increased inactivation time constants at voltages from –20 to +10 mV (Fig. 2*F*, *p* < 0.0001 at –20 and –15 mV, *p* = 0.0185 at –10 mV). Thus, the R1882Q mutation has no effect on hNav1.2 activation but impaired inactivation in HEK cells.

#### The R1882Q mutation increases persistent and resurgent currents

The persistent component of the transient current was measured as the average current amplitude during the last 10% of each depolarization pulse in the activation protocol (Fig. 2*C*, top). The R1882Q mutation increased average persistent current amplitudes and densities at voltages from –65 to +5 mV (Fig. 3*A*,*B*, *p* = 0.0001 from –50 to –25 mV, *p* = 0.0016 at –20 mV, *p* = 0.0526 at –15 mV). When analyzed at a single voltage of –30 mV, the average persistent current amplitude was increased for the R1882Q mutant, compared to WT (WT, –44.97 ± 4.15 pA, R1882Q –100.80 ± 16.53 pA, *n* = 40 WT, *n* = 27 R1882Q; *p* = 0.029). To normalize the persistent current amplitudes to overall expression levels, we also plotted persistent current amplitude as a percentage of the maximum peak transient current amplitude of the cells (value calculated for each individual cell, averages plotted in Fig. 3*C*). This analysis also revealed an increase in persistent current for the R1882Q mutant channel at most voltages from –65 to +5 mV (Fig. 3*C*, *p* < 0.05 from –35 to –20 mV), compared to the WT channel. When the persistent current, as percentage of transient current, was analyzed at –30 mV, the significant increase caused by the R1882Q mutation was again observed (WT 0.57 ± 0.05%, R1882Q 1.15 ± 0.13, *n* = 40 WT, *n* = 27 R1882Q; *p* < 0.0001). Thus, the R1882Q mutation enhances persistent hNav1.2 currents in HEK cells.

Resurgent sodium currents have not been studied with Nav1.2 disease mutations, although they have been associated with other diseases (Jarecki et al., 2010; Hargus et al., 2011; Patel et al., 2016; Tanaka et al., 2016; Xiao et al., 2019). To induce resurgent current through Nav1.2 in HEK cells, the Navβ4 peptide (200 μM) was included in the pipette solution. Cells were depolarized to +30 mV briefly, to open the channel and allow the peptide time to block the central pore, followed by a longer repolarization to voltages ranging from –65 to +10 mV (Fig. 3*D*, top). Resurgent current occurs during the repolarization step, after the transient current is inactivated, when the peptide unbinds and a small inward current occurs between the unbinding of the peptide and the binding of the inactivation particle to the pore. The R1882Q mutation increased average resurgent current amplitudes and densities at voltages from –65 to 0 mV (Fig. 3*D*,*E*, *p* = 0.0082 –0.046 from –35 to –20 mV). When analyzed at a single voltage of –30 mV, the average maximum resurgent current amplitude was increased for the R1882Q mutant, compared to WT [WT –178.40 ± 15.33 pA, R1882Q –284.80 ± 45.71 pA, *n* = 39 WT, *n* = 25 R1882Q; Kruskal–Wallis test, *p* < 0.0001, Dunn’s multiple comparisons test, WT vs R1882Q, *p* = 0.3432 (ns)]. When the resurgent current, as percentage of transient current, was analyzed at –30 mV, the R1882Q mutation still increased the value, compared to WT (WT 2.39 ± 0.18 %, R1882Q 3.35 ± 0.31 %, *n* = 39 WT, 25 R1882Q; *p* = 0.0050). When normalized to the maximum peak transient current amplitudes for individual cells, the resurgent current was, again, increased by the R1882Q mutation at voltages from –65 to –5 mV (Fig. 3*F*, *p* = 0.0053 – 0.0446 from –35 to –20 mV). Thus, the R1882Q mutation enhances hNav1.2 resurgent currents in HEK cells.

### Effects of the R853Q mutation on channel function

#### The R853Q mutation decreases transient current and enhances fast inactivation

**Figure 4.**
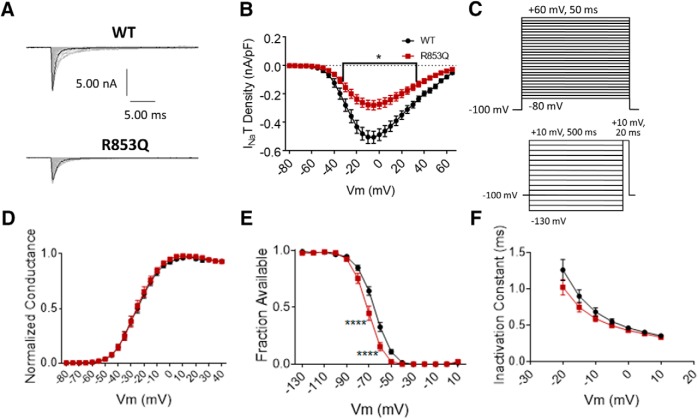
The R853Q mutation reduces transient current size and enhances inactivation. ***A***, Representative raw current trace families elicited by voltage steps from –80 to +60 mV. ***B***, Average transient current densities elicited by voltage steps from –80 to +60 mV (*n* = 40 WT cells, *n* = 16 R853Q cells). ***C***, Voltage stimulation protocols used for collection of activation (top) and inactivation (bottom) data from HEK cells. ***D***, Activation curves. Conductance was calculated as G/G_max_, where G = I/(V_m_-V_rev_), V_rev_ = reversal potential, and G_max_ = maximum inward conductance across all tested voltages (*n* = 41 WT cells, *n* = 16 R853Q cells). ***E***, Inactivation curves. Fraction available was calculated as I/I_max_ for each cell at each voltage step (*n* = 40 WT cells, *n* = 16 R853Q cells). ***F***, Time constants of fast inactivation were calculated by fitting the decay portion of each current trace to a single-exponential equation in PulseFit (HEKA; *n* = 41 WT cells, *n* = 16 R853Q cells). WT data are represented by black circles, and R853Q data by red squares, in ***B***, ***D–F***. Error bars (SEM) are included for every data point in parts ***B***, ***D–F***; please note that many error bars are small and are thus obscured by the symbols. Asterisks indicate significance level (*p < 0.05, ****p < 0.0001). *Figure Contributions*: Emily Mason performed data collection and analysis.

The R853Q mutation decreased the average peak transient current density, compared to WT (Fig. 4*A*,*B*, *p* < 0.05 from –30 to +30 mV), as well as the average maximum peak transient current amplitude (WT –8.75 ± 0.76 nA, R853Q –3.82 ± 0.54 nA, *n* = 41 WT, *n* = 16 R853Q; *p* = 0.0045). This mutation also resulted in a reduction of maximum peak transient current density (WT–0.55 ± 0.05 nA/pF, R853Q –0.28 ± 0.04 nA/pF, *n* = 40 WT, *n* = 15 R853Q; *p* = 0.0033). Thus, the R853Q mutation decreases transient hNav1.2 current amplitudes and densities in HEK cells.

The same voltage protocols that were used to study activation and inactivation in the R1882Q mutant were used to study these parameters for the R853Q mutant (Fig. 4*C*). The R853Q mutation did not significantly alter the voltage-dependence of activation for hNav1.2 channels (in HEK cells; Fig. 4*D*, *p* > 0.99 at all tested membrane potentials). This was supported by a lack of significant difference in average estimated activation midpoints (WT –26.5 ± 1.48, R853Q –25.11 ± 1.81, *n* = 41 WT, *n* = 16 R853Q; one-way ANOVA *p* = 0.79) but it produced a hyperpolarizing shift in both the voltage-inactivation relationship (Fig. 4*E*, *p* = 0.0001 at –70 and –60 mV) and the average estimated inactivation midpoint (WT –65.01 ± 1.27 mV, R853Q –71.46 ± 1.62 mV, *n* = 40 WT, *n* = 16 R853Q; *p* = 0.016). The speed of fast inactivation, measured as a time constant, was unaltered by this mutation (Fig. 4*F*, *p* > 0.99 at all tested membrane potentials). Thus, the R853Q mutation has no effect on hNav1.2 activation but enhances inactivation in HEK cells.

#### The R853Q mutation decreases persistent and resurgent currents

**Figure 5.**
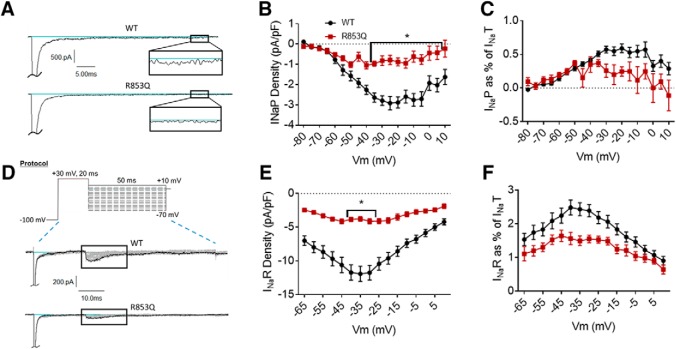
The R853Q mutation reduces persistent and resurgent currents. ***A***, Representative raw current traces from a voltage step to –15 mV. Persistent current (box and inset) was measured as the average current over the last 10% (i.e., 5 ms) of the voltage step. ***B***, Average persistent current densities during the last 10% of voltage steps from –80 to +60 mV (*n* = 39 WT cells, *n* = 16 R853Q cells). ***C***, Average persistent current amplitudes normalized to maximum peak transient current amplitudes (*n* = 40 WT cells, *n* = 16 R853Q cells). ***D***, Resurgent current voltage protocol (top) and representative resulting raw current trace families (middle and bottom). ***E***, Average peak resurgent current densities over a range of voltages from –80 to +60 mV (*n* = 39 WT cells, *n* = 16 R853Q cells). ***F***, Average peak resurgent current amplitudes normalized to maximum peak transient current amplitudes (*n* = 39 WT cells, *n* = 16 R853Q cells). For ***B***, ***C***, ***E***, ***F***, values were calculated for each individual cell and averaged for each group. WT data are represented by black circles, and R853Q data by red squares, in ***B***, ***C***, ***E***, ***F***. Error bars (SEM) are included for every data point in parts ***B***, ***C***, ***E***, ***F***; please note that many error bars are small and are thus obscured by the symbols. Asterisks indicate significance level (*p < 0.05). *Figure Contributions*: Emily Mason performed data collection and analysis.

Persistent current was measured in the same manner for the R853Q mutant channel as for the R1882Q mutant and WT channels. As shown in the representative traces (Fig. 5*A*), the R853Q mutation reduced the average inward persistent current amplitudes and densities between voltages of –60 to +10 mV (Fig. 5*B*, *p* = 0.0028–0.05 from –35 to +5 mV). This reduction in persistent current was further supported by a reduction in the average persistent current amplitude during a –30-mV depolarization (WT –44.97 ± 4.15 pA, R853Q –12.78 ± 4.27 pA, *n* = 40 WT, *n* = 16 R853Q; *p* = 0.0008). When normalized to the maximum peak transient current amplitudes for individual cells, the persistent current was reduced, although non-significantly, by the R853Q mutation, compared to persistent current in the WT channel (Fig. 5*C*, *p* > 0.10 at all tested membrane potentials). This reduction was supported by analysis of the average persistent current during a –30-mV step, as percentage of peak transient current, which showed a significant reduction in this value with the R853Q mutation, compared to WT (WT 0.57 ± 0.05%, R853Q 0.26 ± 0.10, *n* = 40 WT, *n* = 16 R853Q; *p* = 0.037). Thus, the R853Q mutation decreases persistent hNav1.2 currents in HEK cells.

Resurgent current was measured in the same manner for the R853Q mutant channel as for the R1882Q mutant and WT channels (protocol in Fig. 5*D*, top). Compared to cells expressing WT hNav1.2 channels, cells expressing R853Q mutant channels exhibited a significant reduction in average resurgent current amplitudes and densities (Fig. 5*D*,*E*, *p* = 0.0058 – 0.0137 from –40 to –30 mV), which also manifested as a reduction when the maximum peak resurgent current amplitudes were normalized to the maximum peak transient current amplitudes for individual cells (Fig. 5*F*, *p* = 0.0972 at –35 mV, *p* > 0.10 at all tested membrane potentials). These results were supported by analysis of the average resurgent current amplitudes during a –30-mV pulse, which revealed a significant reduction in the average peak resurgent current amplitude (WT –178.40 ± 15.33 pA, R853Q –61.33 ± 7.26, *n* = 39 WT, *n* = 16 R853Q; *p* < 0.0001) and in the average peak resurgent current amplitude expressed as a percentage of peak transient current amplitude (WT 2.39 ± 0.18 %, R853Q 1.55 ± 0.11 %, *n* = 39 WT, 16 R853Q; *p* = 0.0389). Thus, the R853Q mutation decreases resurgent hNav1.2 currents in HEK cells.

#### The R853Q mutation forms a gating pore that passes current at negative membrane potentials

**Figure 6.**
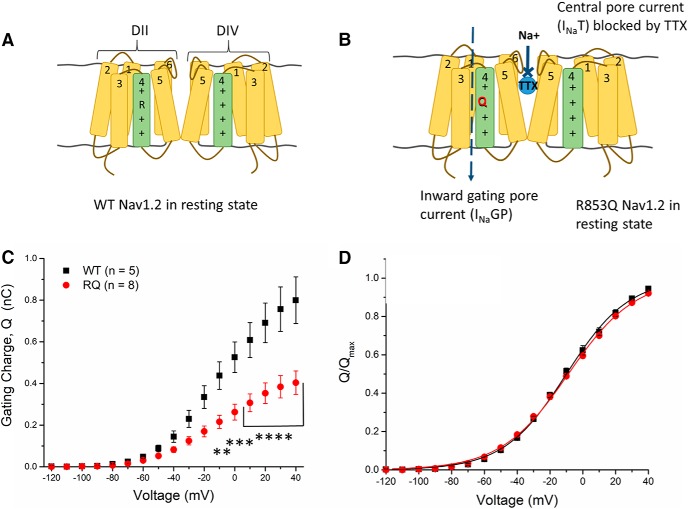
R853Q creates a gating pore and reduces channel surface expression. ***A***, ***B***, Nav1.2 channel in resting state (Domains I and III, N-terminal domain, and C-terminal domain not shown). ***A***, In the resting state, the WT Nav1.2 is essentially impermeable. ***B***, In the resting state, the mutation of arginine 853 to glutamine creates a small aqueous pathway that is blocked when the channel transitions to an open state. Since this channel (i.e., the gating pore) is structurally distinct from the central pore of Nav1.2, current through it can be isolated by blocking the central pore with TTX. ***C***, Average gating charge (Q) values measured from oocytes expressing either WT (black squares) or R853Q (red circles) hNav1.2. The Q_max_ for each group was calculated as the average Q value at +40 (*n* = 5 WT cells, *n* = 8 R853Q cells). ***D***, Voltage dependence of gating charge movement is shown as a normalized gating charge-voltage relationship (*n* = 5 WT cells, *n* = 8 R853Q cells). Average gating charge (Q) across a range of membrane potentials were normalized to the average Q_max_ value for each group. WT data are represented by black squares, and R853Q data by red circles, in ***C***, ***D***. Error bars (SEM) are included for every data point in parts ***C***, ***D***; please note that many error bars are small and are thus obscured by the symbols. Asterisks indicate significance level (**p < 0.01, ***p < 0.001, ****p < 0.0001). *Figure Contributions*: Fenfen Wu performed data collection and analysis.

All of our data up to this point is consistent with the hypothesis that the R853Q mutation confers a loss of function on the hNav1.2 channel. Since the seizures and choreoathetosis (a specific form of irregular involuntary movements) associated with this mutation suggest a possible neuronal gain of function effect, we hypothesized that this mutation increases channel activity via the formation of a gating pore conductance. Such a pore, which is structurally distinct from the central pore, can allow some monovalent cations to leak into the cell through the Domain II voltage sensor, which contains the R853Q mutation (Fig. 6*A*,*B*).

To assess the effects of the R853Q mutation on the gating pore current, cells expressing WT or R853Q mutant hNav1.2 were treated with TTX and subjected to a series of voltage steps. Such experiments in HEK cells yielded inconsistent results which were difficult to interpret. Since *Xenopus* oocytes are much larger and can express more copies of the transfected Nav channel, the peak current amplitude in oocytes is much larger (∼1000–10,000×) than that recorded from HEK cells. We reasoned that because, in oocytes, all currents, including gating pore current, are amplified, this amplification would allow rigorous quantification of any differences in gating pore current amplitude between WT and R853Q mutant hNav1.2 channels. The optimized hNav1.2 WT and R853Q mutant channel constructs were subcloned from the pcDNA3.1 vector into a pGEMHE vector that could be used for sodium channel expression in oocytes. To estimate relative plasma membrane expression levels of WT and R853Q mutant hNav1.2 channels, the average maximal gating charge (Q_max_) of oocytes expressing WT or R853Q mutant hNav1.2 channels was measured in the presence of TTX. The Q_max_ was significantly reduced by the R853Q mutation, compared to WT (Fig. 6*C*, WT 0.85 ± 0.12 nC, *n* = 5; R853Q 0.44 ± 0.06 nC, *n* = 6; *p* < 0.0001 at all voltages positive to 0 mV). The magnitude of this reduction of Q_max_ (∼50%) suggests that the plasma membrane expression level of the channels is reduced by the R853Q mutation, consistent with the reduced amplitude of the transient inward sodium current in HEK cells. Normalizing the Q(V) curves to Q_max_ for each cell and plotting the average across cells revealed no significant change in the midpoint or slope of the gating charge-voltage relationship between WT-expressing and R853Q-expressing oocytes (Fig. 6*D*). This suggests that there is no significant difference in the voltage dependence of total gating charge movement between WT and R853Q mutant channels.

**Figure 7.**
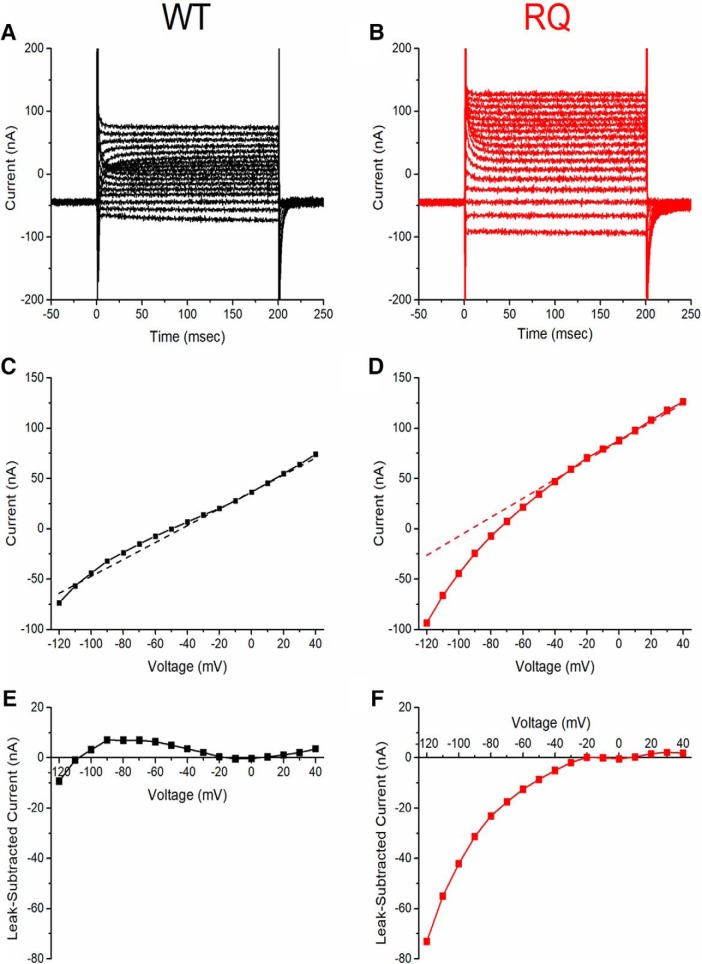
R853Q creates nonlinear leak current. Single-cell representative data (*n* = 1 WT, *n* = 1 R853Q). ***A***, ***B***, Representative raw leak current traces measured from oocytes expressing either WT (black) or R853Q (red) hNav1.2 show inward rectification at hyperpolarized potentials for R853Q that was not observed for WT hNav1.2. Voltage steps over a range from –120 mV to +40 mV were applied from a holding potential of –100 mV. ***C***, ***D***, Leak I-V relationships of the representative cells. Dashed line shows the background linear leakage current, estimated by fitting a line to the steady-state IV plot in the range –10 to +10 mV. Notice that the holding current at –100 mV is entirely from the linear nonspecific leak in WT, whereas for the representative R853Q oocyte the holding current is almost entirely from the contribution of the non-linear inward rectifying component. ***E***, ***F***, Subtraction of the linear background current reveals strong inward rectification for R853Q (red) and low-amplitude nonspecific variance for WT (black). WT data are shown in black (***A***, ***C***, ***E***); R853Q data in red (***B***, ***D***, ***F***). *Figure Contributions*: Fenfen Wu performed data collection and analysis.

**Figure 8.**
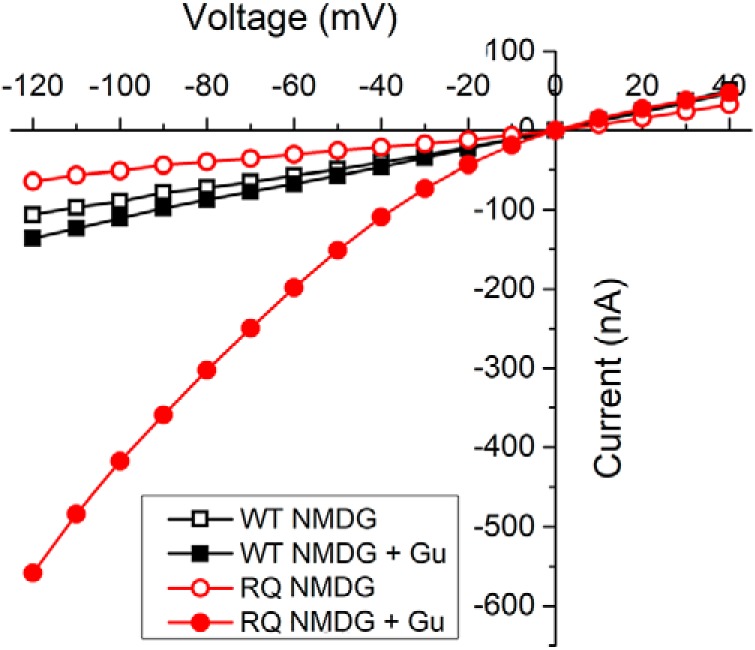
R853Q-induced gating pore is permeable to guanidinium. Single-cell representative data (*n* = 1 WT, 1 R853Q). Leak currents in oocytes expressing either WT (black squares) or R853Q (red circles) hNav1.2 in the absence (empty symbols) and presence (filled symbols) of guanidinium (Gu), a cation believed to be permeant to the gating pore created by the R853Q mutation in hNav1.2. *Figure Contributions*: Fenfen Wu performed data collection and analysis.

To measure gating pore current, the total leak current in oocytes expressing WT or mutant hNav1.2 was measured over a range of membrane potentials in the presence of TTX, which blocked the central pore and prevented transient current conduction (–120 to +40 mV; Fig. 7*A–D*). to isolate the gating pore current, the average nonspecific leak current was subtracted from the total leak current. The average nonspecific leak was estimated by fitting the steady-state current−voltage (I-V) curve (range –20 to +10 mV, end of 200-ms pulse) with a line (Fig. 7*C*,*D*, dotted lines) and subtracted from the total leak current (Fig. 7*C*,*D*, data points) to generate gating pore current I-V curves for WT-expressing and R853Q-expressing oocytes (Fig. 7*E*,*F*). Compared to WT-expressing oocytes, R853Q-expressing oocytes demonstrated an inwardly-rectifying current that diverges from the nonspecific leak current at hyperpolarized potentials (Fig. 7*E*,*F*). We attribute this anomalous inward-rectifying current to the formation of a gating pore in the mutant channel, as supported by dramatically increased current amplitude in the presence of extracellular guanidinium (Fig. 8), as previously reported for gating pore currents in R/X mutations of S4 in Nav1.4 (Sokolov et al., 2010).

**Figure 9.**
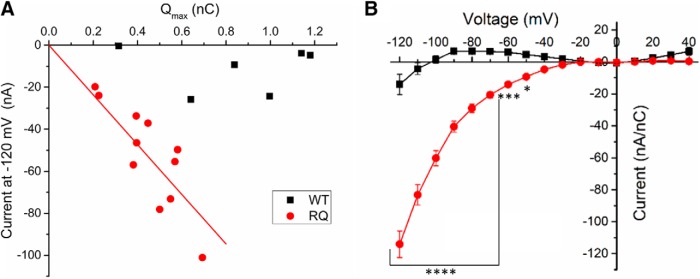
R853Q creates gating pore current. ***A***, Distance of gating charge movement (Q_max_) was plotted against the gating pore current amplitude measured at –120 mV for each cell (*n* = 6 WT cells, *n* = 11 R853Q cells). ***B***, Average leak-subtracted (i.e., gating pore) current was normalized to Q_max_ for each cell and the normalized gating pore I-V relationships for WT (black squares) and R853Q (red circles) were plotted (*n* = 6 WT cells, *n* = 11 R853Q cells). Error bars (SEM) are included for every data point in part B; please note that many error bars are small and are thus obscured by the symbols. Asterisks indicate significance level (*p < 0.05, ***p < 0.001, ****p < 0.0001). *Figure Contributions*: Fenfen Wu performed data collection and analysis.

To further confirm that the inwardly-rectifying current in the R853Q mutant channels is conducted by the gating pore, and not an endogenous conductance, the Q_max_ for each cell was plotted against the measured gating pore current at –120 mV. This plot (Fig. 9*A*) revealed a linear correlation between inward current amplitudes and the corresponding maximal gating charge displacement in individual oocytes for the R853Q mutant channel, but not for the WT channel. This suggests that a gating pore current that is dependent on channel density is present in the R853Q mutant form of hNav1.2 but essentially absent in the WT form.

To facilitate the comparison of gating pore currents in oocytes with different levels of channel expression on the plasma membranes, leak-subtracted currents were normalized to Q_max_ for each oocyte. The average normalized I-V relationships of WT-expressing and R853Q-expressing oocytes revealed a substantial increase in hyperpolarization-activated inward current in R853Q-expressing oocytes compared to WT-expressing oocytes (Fig. 9*B*). These data provide strong support for the theory that a gating pore current exists in the R853Q mutant channel at membrane potentials negative to –30 mV.

## Discussion

Mutations in neuronal voltage-gated sodium channel isoforms are increasingly being identified as underlying otherwise idiopathic cases of epilepsy. Although over 150 different mutations in the Nav1.2 gene, *SCN2A*, have been reported as likely causes of epilepsy, biophysical characterization of only about two dozen of these mutations have been reported to date. Eleven have been shown to have only gain-of-function effects, six have primarily loss-of-function effects, and five have mixed gain- and loss-of-function effects. However, our study is the first to identify enhanced resurgent currents and gating pore currents as effects of *SCN2A* disease mutations.

Among the Nav1.2 mutations suspected to cause epilepsy in human patients, R1882Q and R853Q are two of the most commonly reported mutations (Wolff et al., 2017). The R1882Q mutation has been reported in numerous patients diagnosed with early onset epileptic encephalopathies (6/7 reported onset of 1 d; Carvill et al., 2013; Howell et al., 2015; Trump et al., 2016; Wolff et al., 2017). In contrast, patients with the R853Q mutation were diagnosed with West syndrome or unspecified epileptic encephalopathy, and most or all of these patients had late onsets of seizures (six months to three years); and many exhibit dystonia and/or choreoathetosis (Epi4k Consortium, 2013; Nakamura et al., 2013; Samanta and Ramakrishnaiah, 2015; Kobayashi et al., 2016; Li et al., 2016; Wolff et al., 2017).

### R1882Q mutation effects on hNav1.2 function and predicted effects on neuronal excitability

Four different mutations of the R1882 residue in Nav1.2 have been reported as being putatively pathogenic in epilepsy cases (R1882L, R1882P, R1882G, and R1882Q; Carvill et al., 2013; Baasch et al., 2014; Howell et al., 2015; Schwarz et al., 2016; Trump et al., 2016; Parrini et al., 2017; Wolff et al., 2017). As of yet, the biophysical effects of the R1882L and R1882P mutations have not been studied. The R1882G mutation, which, like R1882Q, neutralizes the positive residue, has been shown to cause gain-of-function effects on Nav1.2 channels in tsA201 cells (Schwarz et al., 2016).

A recent study using Chinese hamster ovary (CHO) cells transiently transfected with hNav1.2 revealed that the R1882Q mutation causes a depolarizing shift in the voltage dependence of fast inactivation, enhances persistent currents, and slows fast inactivation, compared to the WT channel (Berecki et al., 2018). The results of the present study corroborate those findings. Additionally, we show that this mutation substantially enhances resurgent currents in HEK cells, whereas the CHO cell study did not address the possibility of changes in resurgent current due to this mutation. In CHO cells, the R1882Q mutation was also reported to increase peak current density and hyperpolarize the voltage dependence of activation (Berecki et al., 2018), but we did not observe those effects in our experiments. All of the observed changes enhance Nav1.2 channel function, which is predicted to increase neuronal excitability. This prediction was previously tested using a dynamic action potential clamp model. This approach fuses voltage clamp of CHO cells transiently expressing Nav1.2 channels with a computer model of a typical cortical neuron axon initial segment and predict that R1882Q would chronically depolarize neurons and increase their action potential firing activity (Berecki et al., 2018). Importantly, this model system did not incorporate resurgent current mechanisms, which are predicted to further enhance the pro-excitatory impact of the R1882Q mutation.

Resurgent currents have been identified as drivers of both repetitive action potential activity and spontaneous action potential generation (Raman and Bean, 1997; Khaliq et al., 2003; Bant and Raman, 2010; Barbosa et al., 2015; Xiao et al., 2019). Resurgent currents are also enhanced by pro-excitatory disease mutations in other voltage-gated sodium channel isoforms which are associated with pain, myotonia congenital, long-QT syndrome, and *SCN8A* epilepsies (Jarecki et al., 2010; Patel et al., 2016; Xiao et al., 2019). Although ours is the first study to investigate the impact of *SCN2A* mutations on resurgent currents, recordings from DRG neurons demonstrated that Nav1.2 channels can produce resurgent currents in a neuronal background (Rush et al., 2005).

The primary effect of the *SCN2A* R1882Q mutation is impairment of inactivation, which contributes to both increased persistent and resurgent currents (Grieco and Raman, 2004; Theile et al., 2011). It has been shown that the C-terminal domain of Nav1.2 (Mantegazza et al., 2001; Lee and Goldin, 2008; Nguyen and Goldin, 2010), as well as Nav1.3 (Nguyen and Goldin, 2010), Nav1.5 (Cormier et al., 2002; Glaaser et al., 2006), Nav1.6 (Lee and Goldin, 2008; Wagnon et al., 2016), and Nav1.8 (1.5 and 1.8; Choi et al., 2004; Motoike et al., 2004), is involved in modulating fast inactivation. In a recent study using ND7/23 cells, it was shown that the R1872Q mutation in mouse Nav1.6, which is homologous to the hNav1.2 R1882Q mutation, slows inactivation (as evidenced by increased inactivation time constants), hyperpolarizes the voltage dependence of activation, and depolarizes the voltage dependence of inactivation, compared to WT (Wagnon et al., 2016). Since Nav1.2 and Nav1.6 share a high degree of homology in structure and function (amino acid sequences have 75% identity, 85% similarity) homologous mutations in the two isoforms are predicted to have similar functional results. Our results demonstrate that, like the Nav1.6 R1872Q mutation, the R1882Q mutation increased inactivation time constants, indicating a slowing of fast inactivation, and depolarized the voltage dependence of inactivation. In our experiments, the R1882Q mutation also increased persistent currents at physiologically-relevant membrane potentials (amplitude increased significantly from –50 to –20 mV, % of transient current increased significantly from –45 to –25 mV). This effect was seen when R1872 in Nav1.6 was mutated to leucine, but not when it was mutated to glutamine or tryptophan. Additionally, the hNav1.2 R1882Q mutation enhanced resurgent currents, which were not investigated in the study of the mouse Nav1.6 R1872Q mutation. There is substantial evidence suggesting that the C-terminal domain of voltage-gated sodium channels can modulate fast inactivation and persistent currents by interacting with the Domain III/IV linker that contains the inactivation particle (Mantegazza et al., 2001; Motoike et al., 2004; Lee and Goldin, 2008; Nguyen and Goldin, 2010; Wagnon et al., 2016; Clairfeuille et al., 2019). The neutralizing R1882Q mutation may directly or indirectly alter this interaction, thus impairing inactivation and enhancing persistent currents.

### R853Q mutation effects on hNav1.2 function and predicted effects on neuronal excitability

The recent study of *SCN2A* mutations expressed in CHO cells reported that the R853Q mutation in human Nav1.2 decreases peak transient current density and produces a hyperpolarizing shift in the voltage dependence of inactivation (Berecki et al., 2018); these observations were corroborated in our present study using HEK cells. Our data also suggested that this mutation reduces persistent and resurgent currents. The reductions in Q_max_ in oocytes and in current density in HEK cells resulting from this mutation, compared to the WT channel, suggest that this mutation reduces the surface expression of the channel. All of these are loss-of-function effects, which are predicted to decrease neuronal excitability. Although loss of Nav1.2 function is common among late-onset *SCN2A* epilepsies, the choreoathetosis associated with the R853Q mutation suggests an underlying neuronal hyperexcitability. The only proposed effect of this mutation that represents an anomalous channel function and, thus, explains how it may cause neuronal hyperexcitability, is the creation of a gating pore that conducts cationic current. Typical neuronal resting membrane potential is around –70 mV, a membrane potential at which the gating pore in R853Q mutant channels is conducting. The inward cationic current, although small, may chronically depolarize the affected neurons, making them hyperexcitable. Multiple studies have shown that mutations of the highly conserved voltage-sensing residues in the second domain of Nav channels result in gating pore currents (Sokolov et al., 2005, 2007, 2008, 2010; Struyk and Cannon, 2007). Patch clamp data and molecular dynamic simulations have shown that mutation of the second voltage-sensing arginine residue in the bacterial channel, which is homologous to hNav1.2 R853, creates a distinct gating pore that is open in the resting state channel conformation (Gamal El-Din et al., 2014; Jiang et al., 2018). In rat Nav1.2, the R853Q mutation, when paired with the R850Q mutation, produced an inward gating pore current in *Xenopus* oocytes, but the R853Q mutation alone did not produce an observable gating pore current that was significantly different from that of the WT rat channel (Sokolov et al., 2005). This may indicate a species difference in susceptibility. The R853Q mutation in hNav1.2 did not produce an observable gating pore current in CHO cells (Berecki et al., 2018); however, this is likely due to the low current amplitude and density of R853Q channels in CHO cells. We obtained robust expression of hNav1.2 channels in *Xenopus* oocytes and observed substantial gating pore currents with the hNav1.2 R853Q construct. The gating pore current amplitude is linearly correlated with the gating charge (Fig. 9*A*), and the slope of the linear increase is –118.0 ± 8.8 nA/nC (*n* = 11). By comparison, for the gating pore current observed with Nav1.4 in hypokalemic periodic paralysis mutants, the slopes range from 50 to 150 nA/nC (Struyk and Cannon, 2007; Mi et al., 2014). This indicates that the severity of the gating pore leak induced by R853Q in hNav1.2 is comparable to those observed with Nav1.4 disease mutations.

Inward gating pore currents resulting from the mutation of one of the first two arginines in DIIS4 (in hNav1.2, R850 or R853) are predicted to destabilize and even chronically depolarize the membrane potential of affected neurons. Gating pore currents may also disrupt intracellular ion homeostasis, allowing excess sodium ions and possibly even protons to leak into affected neurons through the gating pore (Struyk and Cannon, 2007; Sokolov et al., 2007). Although the generation of gating pore current adds an aberrant function to the hNav1.2 channel, the impact of this anomalous current may depend on the cell background. A depolarizing influence, such as excess sodium entry into the cell, could increase the activity of some neurons by bringing the membrane potential closer to the action potential firing threshold, while paradoxically decreasing excitability in others if the depolarization of the membrane potential reduces sodium channel availability due to the accumulation of inactivated channels. The other effects of the R853Q mutation confer a moderate loss of function on hNav1.2, which is predicted to contribute to decreased neuronal excitability. The dynamic action potential clamp experiments using CHO cells transiently expressing the Nav1.2 channel used a computer model of a typical cortical neuron axon initial segment (Berecki et al., 2018), predicting that the R853Q mutation would decrease action potential firing activity and thus reduce overall neuronal excitability. However, the dynamic clamp model did not consider the possibility that gating pore currents may be induced by this mutation, and that these currents could alter neuronal activity directly, by reducing the action potential threshold, or indirectly, by chronically impacting ion homeostasis and energy metabolism in neurons. Such alterations could cause variable and/or chronic changes in neuronal properties, and these changes may not be readily apparent using dynamic clamp or traditional electrophysiological analyses in heterologous expression systems. Moreover, seizures in patients with R853Q are refractory to AEDs that block the conventional Na-conducting pore, consistent with a pathomechanism based on the gating pore current.

Although the mechanism by which the R853Q mutation causes the observed clinical phenotype is still unclear, it is clearly a pathogenic mutation. Mutations in the homologous residue in three other Nav isoforms (Nav1.1, 1.4, and 1.5) have been shown to be associated with disorders of excitability. In addition to R853Q, ten other neutralizing mutations of seven different voltage-sensing residues in Nav1.2 have been implicated in over 30 patients with epilepsy syndromes. Three mutations in domains I and III of hNav1.2 have been studied *in vitro* (R223Q, R1312T, and R1319Q). Of these three, one (R1312T, in DIII) also exhibits a loss-of-function hyperpolarizing shift in fast inactivation, compared to WT, but R1312T also exhibited multiple gain-of-function effects that were not seen in our experiments with the R853Q mutant (Lossin et al., 2012). The other two (R223Q, R1319Q) were shown to have the opposite effect, exhibiting a depolarizing shift in the voltage dependence of fast inactivation (Scalmani et al., 2006; Misra et al., 2008). The R1319Q mutation, like R853Q, has been shown to reduce the current density of Nav1.2; and the R1319Q mutation was also shown to reduce the surface expression of the channel (Misra et al., 2008). Clinically, the R853Q phenotype most resembles that of R1312T, causing severe seizures that are often refractory to antiepileptic medications, having a late onset (typically more than six months), and being accompanied by severe intellectual disability (Shi et al., 2009; Epi4k Consortium, 2013; Nakamura et al., 2013; Samanta and Ramakrishnaiah, 2015; Kobayashi et al., 2016; Li et al., 2016; Wolff et al., 2017).

Only one other voltage-sensing residue in Domain II of Nav1.2, R856, has been reported to have mutations implicated in epilepsy (Howell et al., 2015; Moller et al., 2016; Wolff et al., 2017). The R856L mutation has been identified in one patient who was diagnosed with epilepsy of infancy with migrating focal seizures (Howell et al., 2015), and the R856Q mutation was identified in two patients diagnosed with Ohtahara syndrome (Moller et al., 2016; Wolff et al., 2017). Neither mutation has been studied in the laboratory, as of yet. Seizure onset was reported to be early (1–2 d) in two of the cases, representing both mutations (Howell et al., 2015; Wolff et al., 2017). At least one child with the R856Q mutation was refractory to antiepileptic medicines and died at the age of three months (Moller et al., 2016; Wolff et al., 2017). Since R853Q and R856Q are mutations of adjacent voltage-sensing residues in the functional Nav1.2 protein, the proximity of these two residues and the severe, refractory epileptic phenotypes caused by both suggests that these mutations may have similar effects on the structure, and thus also the function of the channel. We predict that several, if not all, of these other S4 mutations in hNav1.2 may also induce gating pore currents.

### Applications

The primary goal of this study was to characterize the functional effects of two missense mutations on the hNav1.2 channel, to develop a better understanding of the mechanisms by which ion channel mutations lead to seizure disorders. The various profiles of biophysical functional changes caused by the epileptogenic *SCN2A* mutations that have been studied *in vitro* suggests that distinct missense mutations in hNav1.2 have different mechanisms of seizure generation. For example, R853Q and R1882Q both lead to severe epilepsy, although with distinct clinical phenotypes (Carvill et al., 2013; Epi4k Consortium, 2013; Nakamura et al., 2013; Howell et al., 2015; Samanta and Ramakrishnaiah, 2015; Kobayashi et al., 2016; Li et al., 2016; Trump et al., 2016; Wolff et al., 2017); and, in this study, these mutations have opposite effects on hNav1.2 persistent current, resurgent current, and voltage dependence of inactivation. Given that distinct missense mutations in the same ion channel generate seizures by unique molecular mechanisms, individual mutations may require targeted pharmacotherapeutic strategies to normalize the channel activity and prevent seizure generation (epileptogenesis). For example, epileptic patients with a mutation that increases persistent current as the primary pathologic effect (e.g., A263V; Parrini et al., 2017) would benefit from a pharmacological compound that selectively inhibits persistent current; while the same compound would likely be ineffective in patients with mutations generating seizures through a loss of channel expression (e.g., R102X; Kamiya et al., 2004). An ideal pharmacotherapeutic strategy for any epileptic patient with a pathogenic *SCN2A* mutation may involve selectively targeting the specific pathologic biophysical effects of each mutation, which seem to often include the enhancement of aberrant currents such as persistent, resurgent, and gating pore currents. Such a strategy would maintain or restore healthy channel conductance and gating, as is seen in WT hNav1.2 channels, so as not to produce excessive sedation or other adverse effects in patients. Cannabidiol (CBD) has been demonstrated to preferentially inhibit resurgent current over transient current in hNav1.6 channels expressed in HEK cells, while also blocking the enhancement of persistent current by an epileptogenic hNav1.6 mutation (Patel et al., 2016). Although the primary mechanism of antiepileptic action of CBD is unclear, it is known to antagonize voltage-gated sodium, potassium, and calcium channels; and it has shown some promise as an antiepileptic compound in both preclinical and clinical studies (Jones et al., 2010; Hill et al., 2014; Mao et al., 2015; Devinsky et al., 2016, 2017; Patel et al., 2016; Ghovanloo et al., 2018). In June of 2018, a CBD formulation called Epidiolex (a Schedule I drug produced by GW Pharmaceuticals; Greenwich Biosciences, Inc., 2018) became the first form of CBD to gain FDA approval, for the treatment of refractory Dravet syndrome and Lennox–Gestaut syndrome (U.S. Food and Drug Administration, 2018; Drugs.com, 2019; Greenwich Biosciences, Inc., 2018). We predict that many of the over two dozen S4 charge neutralizing mutations in Nav1.1, Nav1.2, and Nav1.6 that have been identified in patients with epilepsy also induce gating pore currents that contribute to the disease phenotype. Gating pore currents are unlikely to be targeted by standard clinical therapies and, thus, novel approaches may be needed to ameliorate the impact of the gating pore currents produced by some neuronal sodium channel disease mutations.

Ultimately, we expect that this research will contribute to a knowledge base and resources that will lead to more effective treatments for patients with epileptogenic *SCN2A* mutations. The next step in that process will be screening currently available antiepileptic drugs and novel, putatively antiepileptic compounds in cells expressing mutant hNav1.2 channels, with the aim of discovering the compound that best normalizes the channel activity (to resemble that of WT) for each mutation. The therapeutic value of such compounds could be confirmed in neurons expressing the mutant channels, and the correlation of patient-reported efficacy of currently-available antiepileptic drugs with the effects of the same drugs on mutant channels *in vitro* could be studied. If the correlation is strong, then the *in vitro* studies could provide suggestions of compounds that will likely be efficacious in patients with particular epileptogenic mutations.

## Synthesis

Reviewing Editor: Katalin Toth, Universite Laval Decisions are customarily a result of the Reviewing Editor and the peer reviewers coming together and discussing their recommendations until a consensus is reached. When revisions are invited, a fact-based synthesis statement explaining their decision and outlining what is needed to prepare a revision will be listed below. The following reviewer(s) agreed to reveal their identity: Holger Lerche.

The authors have thoroughly revised their manuscript and addressed all issues raised by the reviewers.

There is only one small details they should address in the revised manuscript:

In several figures the error bars cannot be seen, presumably since they are smaller than symbol size. The authors should mention that in each figure legend. Also the number of experiments should be somewhere included for each graph shown.

## Author Response

Responses to Reviewers:

Comment: In several figures the error bars cannot be seen, presumably since they are smaller than symbol size. The authors should mention that in each figure legend. Also the number of experiments should be somewhere included for each graph shown.

Response: The authors thank the reviewers for reviewing the paper and making these suggestions. The recommended changes have been made in the figure legends.

Change in Manuscript: For each relevant figure, the number of cells in each group is now specified in the corresponding section of the figure legend; and the following statement has been added near the end of each relevant figure legend: “Error bars (SEM) are included for every data point in parts ...; please note that many error bars are small and are thus obscured by the symbols.”

Current Manuscript Lines: 693-773 (legends for figures 2, 3, 4, 5, 6, and 9)
